# Obstructive Sleep Apnea Screening Among Hypertensive Patients in Low-Resource Settings

**DOI:** 10.7759/cureus.79710

**Published:** 2025-02-26

**Authors:** Sasa Dragic, Vesna Dragosavac, Suzana Savic, Danica Momcicevic, Biljana Zlojutro, Milka Jandric, Tijana Kovacevic, Vlado Djajic, Pedja Kovacevic

**Affiliations:** 1 Medical Intensive Care Unit, University Clinical Centre of the Republic of Srpska, Banja Luka, BIH; 2 Family Medicine, Public Health Institution "Dr. Mladen Stojanović", Laktasi, BIH; 3 Family Medicine, Public Health Institution "Health Center" Banja Luka, Banja Luka, BIH; 4 Neurology, University Clinical Centre of the Republic of Srpska, Banja Luka, BIH

**Keywords:** low resource countries, obstructive sleep apnea (osa), sleep apnea monitoring, sleep medicine specialty, systemic arterial hypertension

## Abstract

Introduction: In low-resource countries, where sleep medicine centers are limited, screening tools are essential for identifying individuals at risk for obstructive sleep apnea (OSA). This study aimed to identify additional factors that, in combination with the Berlin Questionnaire (BQ) and the Epworth Sleepiness Scale (ESS), could improve the identification of hypertensive patients requiring further evaluation.

Subjects and methods: This cross-sectional study was conducted at the Family Medicine Service of the Public Health Institution "Dr. Mladen Stojanovic" in Laktasi, Bosnia and Herzegovina, which serves approximately 35,000 inhabitants. Using a questionnaire with defined important parameters alongside screening tools - BQ and ESS - the study aimed to present descriptive indicators of the observed population and to determine identifiers of sleep disorders (primarily OSA) using the chi-square test.

Results: A total of 500 patients with arterial hypertension (254 females and 246 males; median age 60 years (IQR: 50-68 years) and median body mass index (BMI) 26.9 kg/m² (IQR: 24.8-29.8 kg/m²)) were screened using the BQ and the ESS. High risk for sleep disorders was identified in 25.4% of subjects using the BQ and in 25% using the ESS. Smoking, alcohol consumption, a history of causing a traffic accident, and the use of sleeping pills were correlated with positive results on both screening tests.

Conclusion: Patients with arterial hypertension who smoke, consume alcohol regularly, have a positive history of causing a traffic accident, and use sleep medications may benefit from screening for OSA and consideration for referral to sleep medicine centers.

## Introduction

Obstructive sleep apnea (OSA) is the most common of all sleep-related breathing disorders. It is characterized by repeated interruptions of breathing during sleep due to apnea and/or hypopnea caused by the collapse of the upper airways. This leads to gas exchange disorders and disrupted sleep that does not provide restorative rest [1]. In addition to disturbing the quality of breathing during sleep, OSA also results in decreased daily efficiency and, ultimately, dysfunction of various organ systems, primarily the cardiovascular system [2]. The link between OSA and cardiovascular diseases can be attributed to the fact that occasional episodes of hypoxemia and hypercapnia lead to increased sympathetic activity, heart rate variability, and arterial tension, and consequently, induce the production of vasoactive substances (catecholamines) and inflammatory mediators (interleukin-6 (IL-6), interleukin-8 (IL-8), interleukin-1 beta (IL-1β), tumor necrosis factor-alpha (TNF-α), macrophage inflammatory protein-2 (MIP-2), cyclooxygenase-2 (COX-2), and C-reactive protein (CRP)) [3,4]. This leads to oxidative stress, endothelial dysfunction, insulin resistance, thrombosis, and changes in intrathoracic pressure [5]. The extremely negative intrathoracic pressure due to repeated forced inspirations caused by partially closed airways increases the transmural gradient across the atrium, ventricle, and aorta, which disrupts ventricular function and causes hemodynamic instability. These effects can also impact the central nervous system, particularly in patients with compromised cerebrovascular circulation [3].

Overall, the prevalence of OSA in the middle-aged population is 24% to 26% in men and 17% to 28% in women. Its prevalence is higher in individuals with hypertension, ranging from 30% to 80% [6-8].Approximately 50% of patients with OSA have elevated blood pressure, especially at night; the same is true for various types of arrhythmias, coronary disease, and cardiac decompensation. Additionally, OSA is associated with an increased risk of cerebrovascular disease, type 2 diabetes, and gastroesophageal reflux disease (GERD) [9]. It is estimated that more than 85% of individuals with clinically significant and potentially treatable OSA have never been diagnosed [10]. If left untreated, OSA can progress over time [11], and sleep medicine has experienced expansion in recent years. Most European countries (such as Germany, Spain, and France) have incorporated polysomnography into routine medical examinations for potential drivers [12], while in neighboring countries (such as Serbia), polysomnography is mandatory for professional drivers. Sleep disorders, particularly OSA, are still not sufficiently recognized in the Republic of Srpska and Bosnia and Herzegovina, despite their frequency and severe consequences. Based on the literature analysis by Benjafield et al. and using the algorithm developed by Duce et al., it is estimated that the number of patients aged 30-69 years with OSA in Bosnia and Herzegovina is 2,185,242 [13,14]. In countries classified as low-resource settings (LRS) [15,16], simple screening tools such as the Berlin Questionnaire (BQ) and the Epworth Sleepiness Scale (ESS) can be valuable in identifying potential patients who should be referred to centers specializing in sleep disorders. Although the sensitivity and specificity favor the BQ, there are recommendations for the combined use of these tests for screening patients with OSA [17,18]. This study was designed to highlight the importance of sleep disorders (primarily OSA) in the population of patients with already diagnosed arterial hypertension in the Republic of Srpska. Regarding the limited number of literature sources, our study objectives have emerged to identify patients with potential sleep disorders using the BQ and the ESS as screening tools and to determine which recorded parameters correlate with positive results from these screening tests (BQ ≥ 2; ESS ≥ 10). To establish the parameters that, when combined with positive results from both screening tests, indicate a high probability of OSA and identify patients with arterial hypertension who should be referred to specialized healthcare for sleep disorder management.

## Materials and methods

Study design

This research was designed as a cross-sectional study. The sample consisted of 500 respondents with arterial hypertension, a chronic cardiovascular disease. The study was conducted between December 1, 2020, and February 1, 2021, at the Family Medicine Service of the Public Health Institution "Dr. Mladen Stojanović in Laktasi, Bosnia and Herzegovina. Approximately 35,000 residents rely on this healthcare facility. The study included patients with arterial hypertension who were scheduled for an examination at the family medicine clinic for conditions or illnesses unrelated to sleep disorders at the time. Sixteen family medicine teams participated in the research, with each team having approximately 2,000 or more registered patients. Data collection took place from 07:00 to 15:00 on working days. The survey was conducted in the waiting rooms of the family medicine clinics with the assistance of nurses/technicians, while family medicine physicians provided final supervision over the completed questionnaires. This research was approved by the Ethics Committee of the Public Health Institution "Dr. Mladen Stojanović" Laktasi, Republic of Srpska, Bosnia and Herzegovina (Decision No. 03-EO/20 dated November 20, 2020). Each respondent received a copy of the informed consent form, signed the consent to participate in the study, and completed the questionnaire by hand.

Study questionnaire

The study questionnaire consisted of three sections:

First Section

This part included demographic and general data such as gender, age, length of service, night work, habits, traffic accidents, work-related injuries, sleep problems, medication use, doctor visits due to sleep issues, obesity (expressed through BMI), and snoring during sleep.

Second Section

This part contained the BQ, which included three categories of questions: questions related to snoring and breathing cessation during sleep, questions concerning daytime sleepiness, and questions about comorbidities (arterial hypertension and/or obesity). A positive category was determined if the respondent gave two or more affirmative answers in the first category, the same for the second category, and at least one affirmative answer in the third category (arterial hypertension and/or obesity). If two or all three categories were positive, the respondent was considered to have a high risk for a sleep-breathing disorder [18].

Third Section

This part was the ESS, consisting of eight questions or situations where the respondent might experience varying probabilities of falling asleep (0 - no probability, 1 - low, 2 - moderate, 3 - high). A total score greater than 10 indicated excessive daytime sleepiness [17].

Inclusion criteria

The inclusion criteria for the study were patients with a history of arterial hypertension, aged over 18 years, and signed voluntary consent to participate.

Statistical analysis

After collecting the completed questionnaires, all data were entered into an MS Excel database. Descriptive statistical analysis and statistical inference tests were performed using IBM SPSS Statistics for Windows, Version 26 (Released 2019; IBM Corp., Armonk, New York, United States). The chi-square (χ²) test was applied.

## Results

Patients with a diagnosis of arterial hypertension (n = 500; 254 females and 246 males; median age 60 years (IQR: 50-68 years); median BMI 26.9 kg/m² (IQR: 24.8-29.8 kg/m²)) were screened using the BQ and ESS. After screening, all individuals were found to meet the inclusion criteria for analysis. Table 1 shows the descriptive characteristics of the study participants.

**Table 1:** Descriptive characteristics of the study participants BMI: body mass index; BQ: Berlin Questionnaire; ESS: Epworth Sleepiness Scale

Characteristics	n (%)
Sex	
Male	246 (49.2)
Female	254 (52.8)
Age groups	
18-29 years	4 (0.8)
30-39 years	54 (10.8)
40-49 years	59 (11.8)
50-59 years	118 (23.6)
60-69 years	150 (30)
≥70 years	115 (23)
Working years	
0-10 years	183 (36.6)
11-20 years	82 (16.4)
21-30 years	91 (18.2)
≥31 years	140 (28)
Risk factors and other facts	
Smoking	129 (25.8)
Alcohol abuse	109 (21.8)
BMI (kg/m^2^) ≥ 25	132 (26.4)
Snoring during sleep	254 (50.8)
Working night shifts	90 (18)
Previous traffic accidents	64 (12.8)
Previous work-related injuries	20 (80)
Previous sleep problems	184 (36.8)
Taking sleeping medications	125 (25)
Previous consultation with a sleep specialist	13 (2.6)
Scores	
BQ ≥ 2	127 (25.4)
ESS ≥ 10	125 (25)

Table 2 presents the results of the X^2^ test, which relate defined parameters of importance (gender, age, length of service, night work, habits, traffic incidents, injuries at work, sleep problems, use of medications, visits to the doctor due to sleep problems, obesity expressed through BMI, and snoring in sleep) with BQ and ESS values and parameters that, in combination with a positive result of one or both of the screening tests used, may indicate a high probability of OSA.

**Table 2:** Influence of observed parameters to Berlin Questionnaire (BQ) and Epworth Sleepiness Scale (ESS) BQ: Berlin Questionnaire; ESS: Epworth Sleepiness Scale; BMI: body mass index *: p ≤ 0.05; **: p ≤ 0.01

Parameter	BQ < 2 (n)	BQ ≥ 2 (n)	p-value	ESS < 10 (n)	ESS ≥ 10 (n)	p-value
Sex						
Male	171	75	0.01**	176	70	0.079
Female	202	52		195	55	
Age groups						
18-29 years	4	0	0.528	3	1	0.99
30-39 years	41	13		40	14	
40-49 years	43	16		46	13	
50-59 years	93	25		89	29	
60-69 years	112	38		110	40	
≥70 years	80	35		87	28	
Working years						
0-10 years		43	0.626		5	0.832
11-20 years		25			20	
21-30 years		25			21	
≥31 years		34			33	
Risk factors and other facts						
Smoking	86	44	0.008**	86	43	0.011*
Alcohol abuse	68	41	0.001**	71	38	0.007**
BMI (kg/m^2^) ≥ 25	105	100	0.128	108	101	0.035*
Snoring during sleep	128	126	0.000*	183	71	0.121
Working night shift	68	22	0.818	70	20	0.502
Previous traffic accidents	34	30	0.000**	37	27	0.003**
Previous work-related injuries	68	32	0.09	64	36	0.005**
Previous sleep problems	119	65	0.000**	129	55	0.054
Taking sleeping medications	79	46	0.001**	83	42	0.01**
Previous consultation with a sleep specialist	9	4	0.652	10	3	0.871

## Discussion

Using the BQ and ESS screening tools, 25.4% of respondents with a BQ ≥ 2 and 25% of respondents with an ESS ≥ 10 were identified. Various studies in America and Europe show that one in five adults has some form of OSA, and 1 in 15 has a severe disorder. It is known that more than 15 million people suffer from this disorder in America alone [19]. Although the BQ, compared with the ESS, shows greater sensitivity and specificity in detecting people with OSA in some studies [20], in this study, we obtained approximately the same frequency of results indicating disturbance during sleep. To the best of our knowledge, our study is the only one conducted in Bosnia and Herzegovina that determines the prevalence of OSA in patients with arterial hypertension.

In our study, we had approximately equal gender representation, while other studies have shown that OSA is more common in men than in women, with a male-to-female ratio of approximately 2:1 [21]. More than three-quarters of the respondents belonged to age groups over 50 years old, which is an expected finding, given that these are the age groups that most often seek examination due to certain cardiovascular symptoms, primarily arterial hypertension. The working population dominated (over three-quarters of respondents), and night work, as an indicator of disturbed diurnal rhythm, was found in 18% of respondents, highlighting the importance of this issue in the region where the study was conducted. The interpretation of the results related to habits (smoking and alcohol consumption) should be taken with caution, as these questions are traditionally not always answered honestly. Regarding traffic and work-related injuries, a slightly higher percentage of respondents declared having a work injury (about 20%), while about 12.8% of respondents reported having had a traffic accident at least once in their life. Considering that one of the symptoms of OSA is difficulty in daily functioning and daytime sleepiness, it is expected that this may have certain repercussions on the frequency of traffic and work-related injuries [22]. The National Center for Statistics and Analysis Data reported that, between 2005 and 2009, drowsiness at the wheel caused 1.4% of the six million traffic accidents, and 2.5% of those accidents had a fatal outcome [23]. Also, 36.8% of the respondents stated that they had a problem with sleep, 25% of them reported taking sleeping pills, and only 2.6% of respondents indicated that they had consulted a doctor about sleep problems. This is very alarming data, considering the health consequences that OSA can bring. Literature data also show that more than 85% of people with clinically significant and potentially treatable OSA have never been diagnosed, and the data obtained from this research indicate that this problem is even more pronounced in our population [24]. We found excessive body mass (BMI ≥ 25 kg/m²) in 73.6% of subjects, with no significant gender-related effect on BMI, and given that these subjects were already diagnosed with cardiovascular diseases, it is clear that they represent a high-risk group (high risk for cardiovascular and cerebrovascular events). On the other hand, untreated OSA favors the progression of coronary disease [25], is a risk factor for ischemic stroke [26], and is more common in obese individuals with type 2 diabetes [27]. It is believed that OSA treatment can significantly reduce cerebrovascular risk [28]. Sleep-related breathing disorders are described as part of another entity called obesity hypoventilation syndrome (OHS), which is defined as a combination of obesity (BMI ≥ 30 kg/m²), daytime hypercapnia, and breathing disorders during sleep [29]. Also, in this entity, the same therapeutic measures are applied as in simple OSA [30].

BQ ≥ 2 was significantly more prevalent in men, smokers, people who consume alcohol, individuals who had a traffic accident, individuals who snore, individuals who subjectively stated that they had a sleep problem, and individuals who reported taking sleep medications. Regarding ESS ≥ 10, a significantly higher prevalence was seen among smokers, people who consume alcohol, individuals who had a work injury, people taking sleeping pills, individuals with BMI ≥ 25, and individuals who had a traffic accident. When discussing the gender dimorphism of sleep disorders, certain authors suggest that OSA is more frequently found in men, especially in obese men (the male gender is twice as likely to develop OSA) [19], which was also the case in this study. When observing smoking and alcohol consumption as important factors in the occurrence of sleep disorders, He and colleagues, in their research, confirmed the connection between alcohol consumption and sleep disorders, which correlates with the results of our study. They cited multiple mechanisms, including disruption of the electrophysiological architecture of sleep, triggering insomnia, and influencing the circadian rhythm [31]. Bielicki et al. showed in their research that smokers with more severe OSA have lower mean oxygenation during sleep and greater intensity of daytime sleepiness [32]. In the context of our research, the significance of these data is reflected in the proven connection between smoking and sleep disorders, expressed through the ESS. A study by Terán-Santos et al., conducted in Spain on 102 drivers, showed that the frequency of right-hand traffic accidents in drivers with untreated OSA was six times higher than in the general population. The control group consisted of 152 drivers with polysomnographically excluded sleep disorders [33]. The study by Lin et al. also showed a doubled risk of hospitalization due to traffic trauma in people with OSA [34]. In our study, too, a history of causing a traffic accident was identified as a correlating parameter through the positivity of both screening tools. Earlier research showed that obesity increases the risk of OSA fourfold [19]. In our study, BMI ≥ 25 kg/m² was significantly more prevalent in subjects with ESS ≥ 10 (p = 0.035) than in those with BQ ≥ 2. Snoring during sleep was significantly more prevalent in people with BQ ≥ 2 than in people with ESS ≥ 10, and this finding is justified considering that BQ includes snoring as one of its questions. Snoring is also associated with obesity and morphological characteristics of the neck and is already a known factor associated with the existence of OSA [35].

One of the limitations of the study was the inability to use polysomnography to check whether people with arterial hypertension, who, in addition to the positive results of the screening tools used and defined risk factors, meet the criteria for the diagnosis of OSA (i.e., to check the sensitivity and specificity of this proposed combined method). Additionally, another limitation of the study is the lack of data regarding the history of arterial hypertension (such as the use of antihypertensive medication, average blood pressure level, and the presence of uncontrolled hypertension). The current study identified additional parameters that, in addition to the used screening tools (BQ and ESS), can help identify patients who truly need examination and treatment in specialized sleep medicine centers.

## Conclusions

A high risk for the presence of sleep disorders (primarily OSA), as indicated by the BQ and ESS screening tools, was found in about one-fourth of the subjects with arterial hypertension. Smoking, alcohol consumption, a history of causing a traffic accident, and taking sleeping pills, in combination with BQ ≥ 2 and ESS ≥ 10 results, were identified as indicators of sleep disorders (primarily OSA). In low-resource countries, such as Republika Srpska and Bosnia and Herzegovina, these factors can serve as markers for referring patients to specialized centers for sleep disorders.
